# Maternal Risk Factors, Patterns, and Outcomes of Antenatal Congenital Anomalies: A Hospital-Based Study

**DOI:** 10.3390/diagnostics15101201

**Published:** 2025-05-09

**Authors:** Seham M. Abufraijeh, Ahlam M. Al-Kharabsheh, Ala N. Uwais, Malek Al Qasem

**Affiliations:** Department of Obstetrics and Gynecology, Faculty of Medicine, Mutah University, Alkarak 61710, Jordan; alkharabsheh@mutah.edu.jo (A.M.A.-K.); dr.owais_alaa@hotmail.com (A.N.U.); malikmutah85@gmail.com (M.A.Q.)

**Keywords:** congenital anomalies, antenatal diagnosis, pregnancy outcome, Jordan, maternal-fetal medicine, birth defects

## Abstract

**Background/Objective**: Congenital anomalies (CAs) are structural or functional abnormalities contributing to global neonatal morbidity and mortality. Data on antenatally diagnosed CAs in southern Jordan are limited. The present study reports their prevalence and patterns at the Maternal-Fetal Medicine Clinic of a governmental hospital and examines associated maternal, pregnancy, and delivery outcomes. **Methods**: This retrospective, hospital-based study involved all pregnant women who presented to the clinic between January 2022 to December 2023 and were diagnosed with congenital fetal anomalies. Data about maternal characteristics, classification of fetal anomalies, and pregnancy outcomes were retrieved from medical files. Statistical analyses comprised chi-square tests, Fisher’s exact tests, independent *t*-tests, and multiple binary logistic regressions. **Results**: Among the 750 pregnant women, 74 (9.9%) were diagnosed with CAs. Urinary system anomalies were the most common (54.1%), followed by central nervous system (CNS) anomalies (37.8%). Major anomalies constituted 59.5%, whereas 40.5% were minor anomalies. Gestational age at diagnosis and birthweight were significantly associated with major anomalies (*p* < 0.05). All stillbirths (10.8%) and pregnancy losses before 24 weeks of gestation (9.5%) occurred in cases with major anomalies (*p* < 0.05). Though preterm delivery rates were higher with major anomalies, this association was not statistically significant. **Conclusions:** Major CAs (59.5%) in this southern Jordan cohort were strongly linked to stillbirths and early pregnancy loss, highlighting the need for early diagnosis and improved prenatal care. Targeted interventions, including anomaly scans and risk factor (RF) screening, may reduce the 9.9% prevalence observed.

## 1. Introduction

During the first trimester, the embryo is highly susceptible to internal and external factors that may cause congenital anomalies (CAs) or birth defects [1]. The World Health Organization (WHO) defines CAs as structural or functional deviations present at birth and originating in the prenatal period [2]. These anomalies, classified as major (structural changes with significant medical, social, or cosmetic consequences, often requiring medical intervention) or minor (with minimal health risks and minimal social or cosmetic impacts) [2], affect about 6% of live births worldwide, with prevalence varying by region due to genetic, environmental, and sometimes unknown factors [3,4].

These disorders contribute to an estimated 240,000 neonatal deaths annually within the first 28 days, plus an additional 170,000 deaths in children aged between 1 month to 5 years [3]. In 2015, the Global Burden of Disease study ranked CAs as the fifth leading cause of death in children under five [5]. According to the WHO, a higher proportion of deaths in this age group from birth defects were reported, as other causes of child mortality were successfully controlled [3].

In low- and middle-income countries, where 94% of CAs occur, the reported RFs include poor nutrition, higher infection exposure, and limited healthcare access [3]. Other contributors include chromosomal abnormalities, such as Trisomy 21, maternal infections (syphilis, rubella, and Zika), radiation, nutritional deficiencies (iodine and folate), diabetes, medications (e.g., phenytoin), with advanced maternal age also increasing the risk [3]. While genetic and environmental interactions are implicated, the cause of most CAs remains unknown in 50% of cases [6].

Generally, congenital malformations impose significant social and economic burdens, particularly in resource-limited settings, where treatment costs and social stigma exacerbate challenges [7].

The WHO provides guidelines for CA surveillance to track trends and inform prevention, particularly in resource-limited countries, through its manual “Birth Defects Surveillance” [2]. It defines two types of surveillance: population-based and hospital-based. Our study serves as the foundation for a hospital-based surveillance program, which by definition, includes monitoring pregnancy outcomes affected by CAs in selected hospitals within a specific geographic area [2].

Early prenatal detection is essential for management and reducing stillbirth risk, which rises with multiple anomalies. Son et al. (2021) reported that anomalies in any system increased the odds of stillbirth, with a cumulative effect when multiple anomalies were present [8]. In Jordan, CAs contribute to 13.3% of antepartum and 33.3% of intrapartum stillbirths [9], yet data from southern Jordan are scarce, with one study estimating a 1.28% prevalence among live births [10].

This study is relevant at the regional and national levels as it addresses the lack of CA surveillance in southern Jordan and supporting national efforts to align with WHO guidelines. Globally, it adds to the limited data from low-resource settings, contributing to public health planning and prevention strategies [2].

This study aimed to document the prevalence and patterns of antenatally diagnosed CAs at the Maternal Fetal Medicine Clinic (MFMC) of Al-Karak Hospital, the largest primary referral hospital in southern Jordan. Also, it set out to examine maternal characteristics and pregnancy outcomes based on the type of anomaly with a view to establishing factors independently related to major congenital abnormalities.

## 2. Materials and Methods

This institution-based retrospective study was conducted at Al Karak Governmental Hospital. Data were primarily obtained from the registry of the MFMC, which serves as the official referral center for early morphology scans and anomalous ultrasound findings across southern Jordan, particularly Al-Karak and its surrounding areas. The clinic serves a catchment population of approximately 370,000 to 380,000 residents, providing specialized antenatal care and diagnostic services to a broad regional population. All ultrasound examinations were performed and interpreted by a single MFM specialist with 10 years of clinical experience. Transabdominal ultrasound scans were conducted using a GE P8 system (GE Healthcare, Chicago, IL, USA).

The study included all pregnant women who attended the clinic from 1 January 2022, to 31 December 2023, diagnosed antenatally with fetal CAs, whether booked at the clinic or referred from other facilities. Multiple pregnancies were excluded due to their small sample size and increased anomaly risk.

Fetal CAs were identified via detailed ultrasound and classified per the International Classification of Diseases, Tenth Revision [11]. Specific anomalies were further classified as major or minor based on the following criteria: Ventriculomegaly was considered major if severe (>16 mm) or associated with other anomalies. Isolated mildly affected cases (10–12 mm and 13–15 mm) were classified as minor [12]. Bilateral Hydronephrosis was considered major if severe (>15 mm) or associated with other anomalies. Isolated mildly affected cases (4–6 mm in the second trimester and 7–9 mm in the third trimester) were rated as minor [13]. Unilateral hydronephrosis was classified as major if severe (>15 mm) or associated with other anomalies. Mild isolated cases (4–6 mm in the second trimester and 7–9 mm in the third trimester) were considered minor [13]. Ventricular septal defects (VSDs) were classified as major if large (>3 mm) or associated with other cardiac or noncardiac anomalies [14]. Small isolated VSDs (<3 mm) were considered minor [14].

Data were extracted from the MFMC registry (maternal characteristics, pregnancy details) and labor ward records (delivery outcomes). Maternal variables included age, body mass index (BMI), diabetes history, parity, miscarriages, family history of CAs, and folic acid use. Pregnancy data covered gestational age at diagnosis and CA type/classification. Delivery outcomes included gestational age, birth status (live/stillbirth), fetal sex, and birthweight, recorded on a standardized form.

### 2.1. Statistical Analysis

Categorical data are presented as frequencies and percentages, while continuous data are expressed as means and standard deviations. The chi-square test or Fisher’s exact test was used to examine the association between categorical variables and types of CAs, as appropriate. Mean differences in continuous variables based on the anomaly type were assessed using an independent *t*-test. Additionally, multiple binary logistic regression analyses were performed to identify significant pregnancy outcomes associated with major CAs. Variables with a *p*-value < 0.2 in the bivariate analysis were included in the logistic regression model. Odds ratios (OR) and their corresponding 95% confidence intervals (CI) were reported to indicate the strength of the associations. Statistical significance was set at *p* < 0.05. Data were analyzed using the Statistical Package for the Social Sciences (IBM SPSS Statistics 28; SPSS Inc., Chicago, IL, USA).

### 2.2. Ethical Considerations

The study was approved by the Institutional Review Board of Mutah University (reference number 82024). Patient confidentiality was maintained through coded identifiers and secure data storage; informed consent was waived due to secondary data use.

## 3. Results

Among the 750 pregnant women who received antenatal care at our MFM clinic, 74 (9.9%) were diagnosed with fetal CAs, suggesting that approximately one in 10 cases in our clinic population had a fetal anomaly.

Table 1 describes the frequency of CAs in the various fetal body systems, thereby reflecting the distribution of each condition in the dataset. A total of 103 anomalies were recorded, with syndromic associations excluded, and 74 cases were included in the study. The disparity is due to the presence of multiple anomalies in some patients, meaning a single patient could have had more than one CA.

Urinary system anomalies were the most prevalent, accounting for 54.1% (40/74)of all cases, followed by CNS anomalies, which accounted for 37.8% (28/74) of pregnancies. Major CAs predominated in most systems, notably 93% of CNS and 100% of gastrointestinal anomalies.

Table 2 summarizes the maternal characteristics, pregnancy outcomes, and delivery outcomes of our cohort. The mean age of the patients was 32.2 ± 6.8 years, with a mean BMI of 28.8 ± 4.3 and a mean parity of 2.2 ± 1.8. Only 14.9% used folic acid, 50% had prior miscarriages, and 12.2% had diabetes.

Major CA accounted for 59.5% of cases, with 40.5% minor. Mean gestational age at diagnosis was 26.7 ± 6.6 weeks, with 10.8% stillbirths and 9.5% early pregnancy losses. Preterm delivery before 37 weeks of gestation was observed in 41.8% of the pregnancies.

The analysis in Table 3 explores the relationship between CA type and various maternal and fetal factors. Major CAs were diagnosed earlier (24.2 ± 6.1 vs. 30.4 ± 5.5 weeks, *p* < 0.001) and had lower birthweight (3.0 ± 0.7 vs. 3.2 ± 0.3 kg, *p* < 0.001). All stillbirths and early pregnancy losses occurred with major CAs (*p* **= 0.007** for stillbirths; *p* **= 0.037** for losses <24 weeks), with no cases reported in the minor CA group. Preterm delivery was more frequent in major congenital anomalies (85.7% vs. 14.3%), however, this difference was not statistically significant (*p* > 0.05).

Maternal factors like age, BMI, folic acid use, maternal history of miscarriage, family history of CAs, maternal diabetes, and fetal sex, showed no significant differences, (*p* > 0.05).

Table 4 presents the results of a multiple binary logistic regression (forward conditional selection) evaluating pregnancy outcomes associated with major CAs. Variables identified from bivariate analysis with *p* < 0.20 were included: history of miscarriage (*p* = 0.155), diabetes mellitus (DM) (*p* = 0.146), gestational age at diagnosis (*p* < 0.001), and birthweight (*p* < 0.001). Stillbirths (>24 weeks) and early pregnancy losses (<24 weeks) were excluded due to zero counts in one cell, which resulted in quasi-complete separation and potentially biased estimates.

The final model retained gestational age at diagnosis and birthweight, achieving 73.1% classification accuracy and a Nagelkerke R^2^ of 0.461, indicating that 46.1% of the variance in the likelihood of a major CA was explained by the model.

Gestational age at diagnosis was inversely associated with major CAs, B = −0.124, OR = 0.884, *p* = 0.039, (95% CI: 0.786,0.944), reducing odds by 11.6% per week, reflecting earlier detection of severe cases. Birthweight also showed an inverse relationship, B = −2.896, OR = 0.055, *p* = 0.002, (95% CI: 0.008,0.361), cutting odds by 94.5% per kg, consistent with lower weights in major CAs as shown in Table 4

## 4. Discussion

Among the 750 pregnant women evaluated at our MFMC over two years, 74 (9.9%) were diagnosed antenatally with fetal CAs, indicating a prevalence of approximately one in 10 pregnancies within this referral population. Christianson et al. (2006) estimated a global CA rate of 6% among live births, with Jordan at 73.3 per 1000 live births [15], which is lower than our 98.6 per 1000, likely due to our antenatal focus versus live birth reporting. Similarly Lawn et al. [16] noted that CAs cause less than 10% of stillbirths globally, aligning with our 10.8% stillbirth rate (all major CAs), though higher detection here may reflect the use of advanced diagnostics and the absence of termination options, unlike settings with lower rates (e.g., 2.5% in South Africa) or higher antenatal incidence (e.g., 21% in Ireland) [16].

Our antenatal CA rate of 98.6 per 1000 pregnancies exceeds many published live birth rates, reflecting differences in reporting (antenatal vs. birth prevalence) and our clinic’s role as a referral unit for high-risk cases in southern Jordan. In Saudi Arabia, Kurdi et al. [17] reported a CA rate of 41.2 per 1000 births in 2019, down from 46.5 per 1000 in 2015 [18], attributed to advanced screening, systematic antenatal care, and access to termination for major anomalies. Public awareness campaigns promoting prenatal folic acid use also likely contributed [17], suggesting potential benefits for Jordan, given our high CNS anomaly rate. Regional declines are evident elsewhere: In Qatar, an extended population-based analysis [19] estimated the prevalence at 13 per 1000 live births, a decrease from the 73.4 per 1000 reported by the March of Dimes Foundation in 2006 [15]. Similarly, a study in Egypt estimated the prevalence at two per 1000 live births [20], compared to a previously reported rate of 65.3 per 1000 live births [15].

Breaking down the anomaly patterns, urinary system anomalies were the most common CAs (54%), followed by CNS anomalies (37.8%) and congenital heart disease (CHD) (9%). Similarly Sallout et al. [18], in Saudi Arabia, found genitourinary anomalies predominant (21.28 per 1000 pregnancies), with hydronephrosis leading, either bilateral or unilateral (8.5–8.7 per 1000), and a male bias (53.3% vs. 25% in females). In our cohort, ventriculomegaly was the most frequent CNS anomaly, comprising about 32% of CNS cases (9/28), consistent with Sallout et al. [18], where ventriculomegaly comprising 62.4% of cranial anomalies.

Conversely, our CHD prevalence (9%) is notably lower than Kurdi et al.’s of 36% [17], and a Jordan University Hospital study’s of 34% [21], where fetal echocardiography identified ventricular septal defect (VSD) as the most frequent anomaly (25%). This discrepancy likely reflects our MFMC’s general focus compared to cardiac-specific screening elsewhere, underscoring the importance of targeted diagnostics for CHD detection. Prenatal detection of CHD varies between 25% and 75%, with significant differences observed across different types of anomalies, as detection rates tend to be higher for those involving the four-chamber view and lower for those affecting the outflow tracts [22].

Regarding severity distribution, of 74 antenatal CA cases, 44 (59.5%) were major and 30 (40.5%) were minor, with all stillbirths (10.8%) linked to major anomalies. This high proportion of major CAs likely reflects referral bias, as cases from non-specialist general obstetricians are often reclassified as major after specialist evaluation at our MFMC, and enhanced detection via advanced diagnostics identifies more subtle defects. In Saudi Arabia, Sallout et al. [18] reported an antenatal prevalence of major CAs of 52.17 per 1000 pregnancies, dropping to 46.5 per 1000 live births, suggesting losses similar to our stillbirths. Similarly, Son et al. [8] found major CAs in 4.3% of live births compared to 23.4% of stillbirths, mirroring our severity-outcome pattern. In contrast, the birth prevalence of Qatar was 13 per 1000, which is lower [19], illustrating regional and methodological heterogeneity. Over 90% of clinically significant CAs occur in middle- and low-income settings like Jordan [15], highlighting the burden reflected in our findings.

In terms of sex distribution, our study found 68.9% of all CAs (51/74) in male fetuses (M:F ~2.2:1), with major CAs at 60.8% male (27/44) and minor CAs at 40% male (12/30), though this sex difference was not statistically significant for major anomalies (*p* > 0.05). Ajarmeh et al. [23] similarly documented a male bias in southern Jordan, where 90% of bilateral hydronephrosis cases were male (M:F 1.86:1), although our ratio includes all types of CA, not only urinary anomalies. Verma et al. [6] also reported that urinary defects like multicystic dysplastic kidneys and CHD, such as VSD, are more common in males, aligning with our findings. Historical studies have reinforced the idea of sex-based differences in CAs, with major CA prevalence at 3.9% in males vs. 2.8% in females [24], notably for urinary defects (62% more frequent in males). This suggest that sex appears to influence CA occurrence and type, highlighting the need for further study.

Shifting to risk factors, we categorized risk factors (RFs) by the WHO’s February 2023 congenital anomalies fact sheet [3]. Folic acid intake is crucial for reducing the incidence of neural tube defects (NTDs), which are associated with an increased risk of spontaneous abortion and stillbirth among affected fetuses [25]. Low folic acid intake, a key preventable cause of NTDs, was evident in our cohort, with only 14.9% of mothers (11/74) reporting use. Among non-users (63/74), 61.9% had fetuses with major CAs (39/63) and 38.1% with minor (24/63), though this was not significant (*p* > 0.05). This contrasts sharply with 93% usage in a recent southern Jordan study [26], a 78% gap suggesting a modifiable RF. Globally, folate deficiency drives NTDs, with high-income fortification cutting incidence, while low-income regions like Jordan bear a heavier burden [4,15]. The March of Dimes Global Report on Birth Defects [15] recommends 400 µg/day folic acid, achievable via supplementation or diet, to curb NTDs—relevant given our 37.8% CNS rate. Larger regional studies could confirm this link.

Additionally, diabetes mellitus (DM) affected 12.2% of our CA mothers (9/74), with 33% of these fetuses having major CAs (3/9) and 67% minor (6/9). This may reflect better monitoring reducing severity, aligning with a southern Jordan study showing 4.97% gestational DM, among whom 5.6% had CA infants [27]. Similarly, Kurdi et al. [17] linked DM to 7.3% of CAs in Saudi Arabia (OR 1.98, 95% CI: 1.33–2.95). Given the Middle East’s high DM prevalence, universal gestational DM screening could mitigate CA risk through glycemic control.

The WHO identifies maternal age as an RF for chromosomal anomalies like Down syndrome [3]. In our study, mean maternal age was 33.2 ± 6.4 years for minor CAs and 31.1 ± 7.1 years for major CAs, with no significant difference (*p* > 0.05), aligning with an Al Karak study [10] showing higher CA prevalence at 30–34 years. Other studies show mixed results: Egypt [28] found no age link (2.5% CA prevalence, peak 20–35 years), while a study in the United Arab Emirates [29] noted 9.7% of CAs at ≥35 years without significance, and others [6,30] tied advanced age to aneuploidies.

In the present study, BMI showed no correlation with CA type (*p* > 0.05), unlike Al-Dewik et al. [19], where age ≥35 (OR 3.4) and underweight (OR 7.6) increased chromosomal CA risk, and obesity raised multiple CA odds (OR 3.43).

A family history was found in 8% of our cohort (6/74), with higher proportion among minor anomalies (62.5%) than major (37.5 although this was not significant (*p* > 0.05). This exceeds the 3.3% reported by Abosafi [10] and aligns with regional findings [17,28], although higher rates were seen elsewhere [18]. Family history screening, as recommended by the March of Dimes [15], remains a valuable preventive strategy.

Regarding pregnancy outcomes, this study found gestational age at diagnosis, miscarriage <24 weeks, stillbirths, and low birthweight (LBW) differed significantly between CA types (*p* < 0.05), though only gestational age at diagnosis (OR 0.884, *p* = 0.039) and birthweight (OR 0.055, *p* = 0.002) were associated with major CAs in regression. The association between congenital malformations and LBW has been extensively discussed in the literature. LBW (<2500 g) was more prevalent in major CAs, consistent with studies linking congenital defects to LBW [28,29,31] and Qatar’s OR of 5.88 [19]. Furthermore, LBW is more commonly observed in babies with major congenital abnormalities than in those with minor anomalies [32,33]. Though our study did not look at birthweight by particular anomaly type, previous research has indicated that fetuses with CHD tend to have lower birth-weight, implying a possible link between some anomalies and compromised fetal growth [34].

Preterm delivery (<37 weeks) occurred in 53.6% of major CAs vs. 46.4% of minor, supporting trends in preterm CA rates [28,29,30], though not significant in our final model (*p* > 0.05). All stillbirths (10.8%) were major CAs (*p* < 0.05), aligning with Son et al.’s [8] OR of 4.33 for CA-related stillbirths, with higher risks for anomalies like cystic hygroma (adjusted OR 29.97).

These outcomes highlight major CAs’ impact on fetal viability and growth, mirroring regional patterns.

Recent studies indicate that especially in fetuses with CA, gestational age by itself could not be enough to forecast results in preterm deliveries. Villar et al. suggest a functional and etiologically focused method to more accurately define the preterm birth syndrome and its effects in this group [35].

### 4.1. Strengths

Comprehensive data on antenatal CAs in Jordan are scarce, and previous studies have focused either on neonatal death or specific defects. This hospital-based investigation is the first comprehensive report to deeply analyze maternal and fetal characteristics alongside CA patterns (e.g., 9.86% prevalence, 59.5% major), filling a critical gap. It lays the groundwork for future multicenter research in pursuit of enhancing child health, advancing sustainable development goal 3 [36] through the prevention of avoidable neonatal and under-five mortality due to CAs.

### 4.2. Limitations

This study’s single-center design at Al Karak MFMC limits generalizability to broader Jordanian populations, a constraint that future multicenter studies could address. Additionally, the institutional medical record system separates maternal and neonatal records post-delivery, initially linked under the mother’s name, then updated to the baby’s, complicating outcome tracking, especially for neonatal intensive care unit cases. While capturing many pregnancy outcomes (e.g., 10.8% stillbirths) [2], this restricts comprehensive CA monitoring across the population.

## 5. Conclusions

Our study of 750 pregnant women at Al Karak MFMC revealed a 9.86% antenatal CA rate, with 59.5% classified as major anomalies, indicating a considerable burden on hospital resources. Major CAs were significantly associated with adverse outcomes, including LBW (OR 0.055), stillbirths (10.8%, *p* < 0.05), and higher preterm delivery (53.6%), highlighting the importance of early detection and management. Low folic acid supplementation (14.9%) and the prevalence of maternal diabetes (12.2%) highlight modifiable risk factors, emphasizing the need for strengthened prenatal supplementation and screening programs. In contrast, demographic variables and family history showed no statistically significant associations (*p* > 0.05).

Future studies with larger, more diverse populations are essential to better understand the etiology of CAs and inform prevention strategies. Implementing universal screening and establishing an active surveillance system are critical steps toward accurately mapping CA patterns and prevalence across Jordan.

## Figures and Tables

**Table 1:** The distribution of congenital anomalies across different body systems, *n* = 74.

Body System, ICD-10 *, Total Malformations	Anomaly (ICD-10) *	Major	Minor	Total in System (%)
Central Nervous System, (Q00–Q07), (*n* = 28)	Ventriculomegaly ** (Q04.8)	7	2	9 (32.1%)
	Encephalocele (Q01)	5	0	5 (17.9%)
	Spina Bifida/Meningocele (Q05)	5	0	5 (17.9%)
	Agenesis/Atrophy of Corpus Callosum (Q04.0)	3	0	3 (10.7%)
	Chiari Malformations (Q07.0)	3	0	3 (10.7%)
	Holoprosencephaly (Q04.2)	2	0	2 (7.1%)
	Microcephaly (Q02)	1	0	1 (3.6%)
Urinary, (Q60–Q64), (*n* = 40)	Bilateral Hydronephrosis ** (Q62.0)	8	17	25 (62.5%)
	Unilateral Hydronephrosis ** (Q62.0)	2	7	9 (22.5%)
	Renal Agenesis (Q60.2)	2	0	2 (5%)
	Multicystic Dysplastic Kidney (Q61.4)	2	0	2 (5%)
	Infantile Polycystic Kidneys (Q61.1)	2	0	2 (5%)
Cardiovascular, (Q20–Q28), (*n* = 7)	Cardiomegaly (Q24.8)	4	0	4 (57.1%)
	Ventricular Septal Defect ** (Q21.0)	1	1	2 (28.6%)
	Aortic Stenosis (Q23.0)	1	0	1 (14.3%)
Gastrointestinal and Abdominal wall, (Q38–Q45), (*n* = 9)	Omphalocele, (Q79.2)	6	0	6 (66.7%)
	Gastroschisis (Q79.3)	1	0	1 (11.1%)
	Duodenal Atresia (Q41.0)	1	0	1 (11.1%)
	Esophageal Atresia (Absent Stomach) (Q39.0)	1	0	1 (11.1%)
Syndromic Associations	Meckel-Gruber Syndrome (Q61.9)	3	0	3 (60%)
	Trisomy 18 (Edwards Syndrome) (Q91.3)	1	0	1 (20%)
	Congenital High Airway Obstruction Syndrome (Q31.8)	1	0	1 (20%)
Musculoskeletal System Anomalies, (Q65–Q79), (*n* = 12)	Skeletal Dysplasia (Q77)	6	0	6 (50%)
	Club Foot (Q66.0)	2	1	3 (25%)
	Polydactyly (Q69)	2	1	3 (25%)
Other Anomalies, (*n* = 7)	Hydrops Fetalis (P83.2)	6	0	6 (85.7%)
	Cystic Hygroma (Q18.0)	1	0	1 (14.3%)

Counts represent cases with the specified anomaly present. The balance of cases (out of *n* = 74) for each anomaly represents absence (“No”).* International Classification of Diseases, Tenth Revision (ICD-10) [11]. ** Ventriculomegaly, hydronephrosis (bilateral/unilateral), and VSD were classified as major anomalies if severe or associated with other anomalies. Cases of mild isolation were classified as minor cases.

**Table 2:** Maternal characteristics and pregnancy outcomes, *n* = 74.

Scale Variables	Category	Mean ± SD ^1^
Maternal age		32.2 ± 6.8
Parity		2.2 ± 1.8
BMI ^2^		28.8 ± 4.3
Gestational age at diagnosis, weeks		26.7 ± 6.6
Birthweight, kg		2.9 ± 0.6
**Categorical Variables**	***n*** **(%)**	**Mean ± SD**
Folic acid intake	11 (14.9)	
History of miscarriage	37 (50)	
History of an anomaly in the family	8 (10.8)	
Presence of Diabetes	9 (12.2)	
Fetal gender		
Female	23 (31.1)	
Male	51 (68.9)	
Type of CA ^3^		
Minor	30 (40.5)	
Major	44 (59.5)	
Pregnancy outcome		
Loss <24 weeks	7 (9.5)	
Still birth >24 weeks	8 (10.8)	
Alive	59 (79.7)	
Delivery time, *n* = 67		
Term (>37 weeks)	39 (58.2)	
Preterm (<37 weeks)	28 (41.8)	

For categorical variables, counts (*n*) represent cases present; balance is absence (e.g., 74 − 11 = 63 for no folic acid intake). ^1^ SD: standard deviation, ^2^ BMI: body mass index, ^3^ CA: congenital anomaly.

**Table 3:** Type of congenital anomalies in relation to maternal and fetal factors.

Scale Variable	Type of Congenital Anomaly		Test Value	*p*-Value
	Minor	Major		
Maternal age	33.2 ± 6.4	31.6 ± 7.1	0.960	0.340 ^t^
BMI ^1^	29.2 ± 4.7	28.5 ± 4.1	0.074	0.465 ^t^
Parity	2.2 ± 1.7	2.2 ± 1.8	0.040	0.972 ^t^
Birthweight/kg	3.2 ± 0.3	3.0 ± 0.7	4.830	<0.001 ^*t^
GA ^2^ at CA ^3^ diagnosis	30.4 ± 5.5	24.2 ± 6.1	4.470	<0.001 ^*t^
**Categorical Variable**	**Type of Congenital Anomaly**		**Test Value**	***p*** **-Value**
	**Minor** ***n*** **(%)**	**Major** ***n*** **(%)**		
Folic acid	6 (54.5)	5 (45.5)		0.336 ^F^
History of miscarriage	12 (32.4)	25 (67.6)	2.018	0.155 ^x2^
History of an anomaly in the family	5 (62.5)	3 (37.5)		0.257 ^F^
Presence of Diabetes	6 (66.7)	3 (33.3)		0.146 ^F^
Gender: Female	10 (43.5)	13 (56.5)	0.119	0.730 ^x2^
Male	20 (39.2)	31 (60.8)		
Pregnancy outcome >24 weeks				0.007 *^F^
Alive	30 (50.8)	29 (49.2)		
Stillbirth	0 (0.0)	8 (100.0)		
Pregnancy Loss <24 weeks	0 (0.0)	7 (100.0)		0.037 *^F^
Term delivery (>37 weeks), *N* = 39	26 (66.7)	13 (33.3)	5.406	0.064 ^FH^
Preterm delivery (<37 weeks), *N* = 28	4 (14.3)	24 (85.7)		

For categorical variables, counts represent the “Yes” category; balance is “No” (e.g., 63 − 5 − 6 = 52 for no folic acid use). ^1^ BMI: body mass index, ^2^ GA: gestational age, ^3^ CA: congenital anomaly. ^t^: independent *t*-test, ^X2^ chi-square, ^F^: Fisher exact test, ^FH^, Fisher-freeman-Halton test. * Statistically significant, *p*-value < 0.05.

**Table 4:** Pregnancy outcomes association with major congenital anomalies, *N* = 62.

Variable	B ^1^	S.E. ^2^	*p*-Value	Ors ^3^	95% C.I. ^4^ for (ORs)	
					Lower	Upper
GA ^5^ at diagnosis	−0.124	0.060	0.039	0.884	0.786	
Birthweight (kg)	−2.896	0.957	0.002	0.055	0.008	

^1^ B: Estimated coefficient, ^2^ S. E: standard error, ^3^ ORs: odd ratios, ^4^ C.I: confidence interval; ^5^ GA: gestational age.

## Data Availability

The original contributions presented in this study are included in the article. Further inquiries can be directed to the corresponding author.

## Abbreviations

The following abbreviations are used in this manuscript:

BMI	Body Mass Index
CA	Congenital Anomaly
CAs	Congenital Anomalies
LBW	Low Birth Weight
OR	Odds Ratio
CI	Confidence Interval
RF	Risk Factor
WHO	World Health Organization
NTD	Neural Tube Defect
CNS	Central Nervous System
CHD	Congenital Heart Disease
DM	Diabetes Mellitus
